# Supplementary material for: Exploring the structure and dynamics of peptide nanodiscs through a synergistic approach with NMR spectroscopy, SAS and MD simulations
Source: Commun Chem. 2026 Apr 9;9:196. doi: 10.1038/s42004-026-02015-5 (PMC13247028; doi:10.1038/s42004-026-02015-5)
Supplement: Supplementary file 2 — Supplementary Information [file 42004_2026_2015_MOESM2_ESM.pdf]

Supplementary Information for

Exploring the structure and dynamics of peptide nanodiscs through a synergistic approach with NMR spectroscopy, SAS and MD simulations

Sirine Nouri,*[1] Akseli Niemelä,[1] Ricky Nencini,[1,2] Georgios Kolypetris,[1] Tuomas Niemi-Aro,[2] Salla I. Virtanen,[2] O. H. Samuli Ollila,[2,3] and Artturi Koivuniemi,*[1]

[1]Division of Pharmaceutical Biosciences, Faculty of Pharmacy, University of Helsinki, Helsinki, Finland
[2]Institute of Biotechnology, University of Helsinki, Helsinki, Finland
[3]VTT Technical Research Centre of Finland, Espoo, Finland
*Corresponding author

Email: sirine.nouri@helsinki.fi, artturi.koivuniemi@helsinki.fi

This PDF file includes:

Figures S1 to S9
Tables S1 to S4

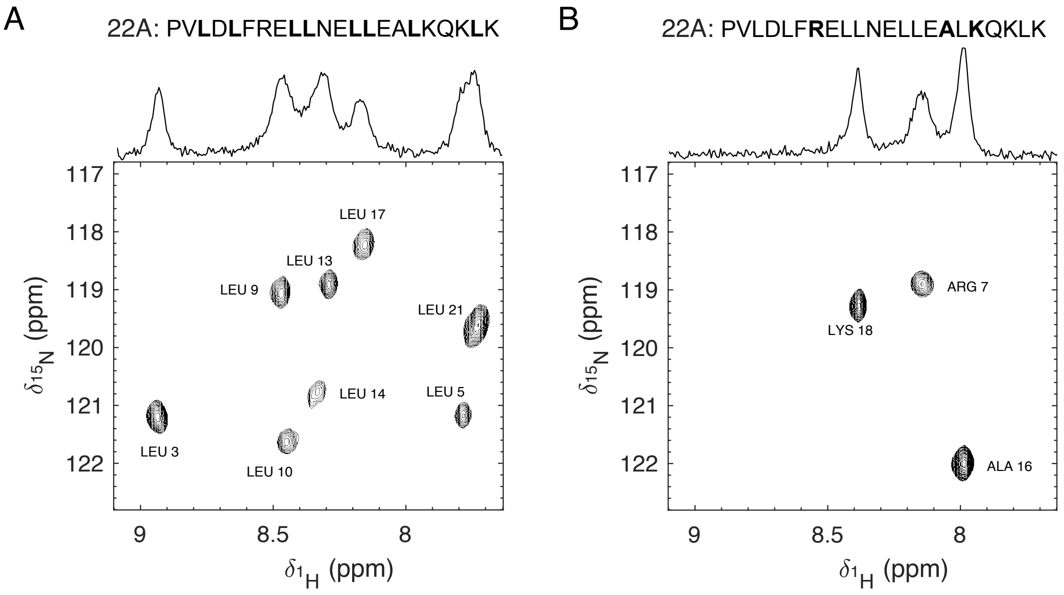

Figure S1. ^{1}H-^{15}N HSQC NMR spectra of peptide nanodiscs. (A) [L-^{15}N] labeled sample. (B) [R_7-A_{16}-K_{18}-^{15}N-^{13}C] labeled sample. 1D projections are shown on top of the spectra. Sequence of 22A peptide with labeled residues in bold and assignment are indicated. Spectra were measured at 850 MHz in 1/50 X PBS buffer (pH 7.4) with 10% D_2O.

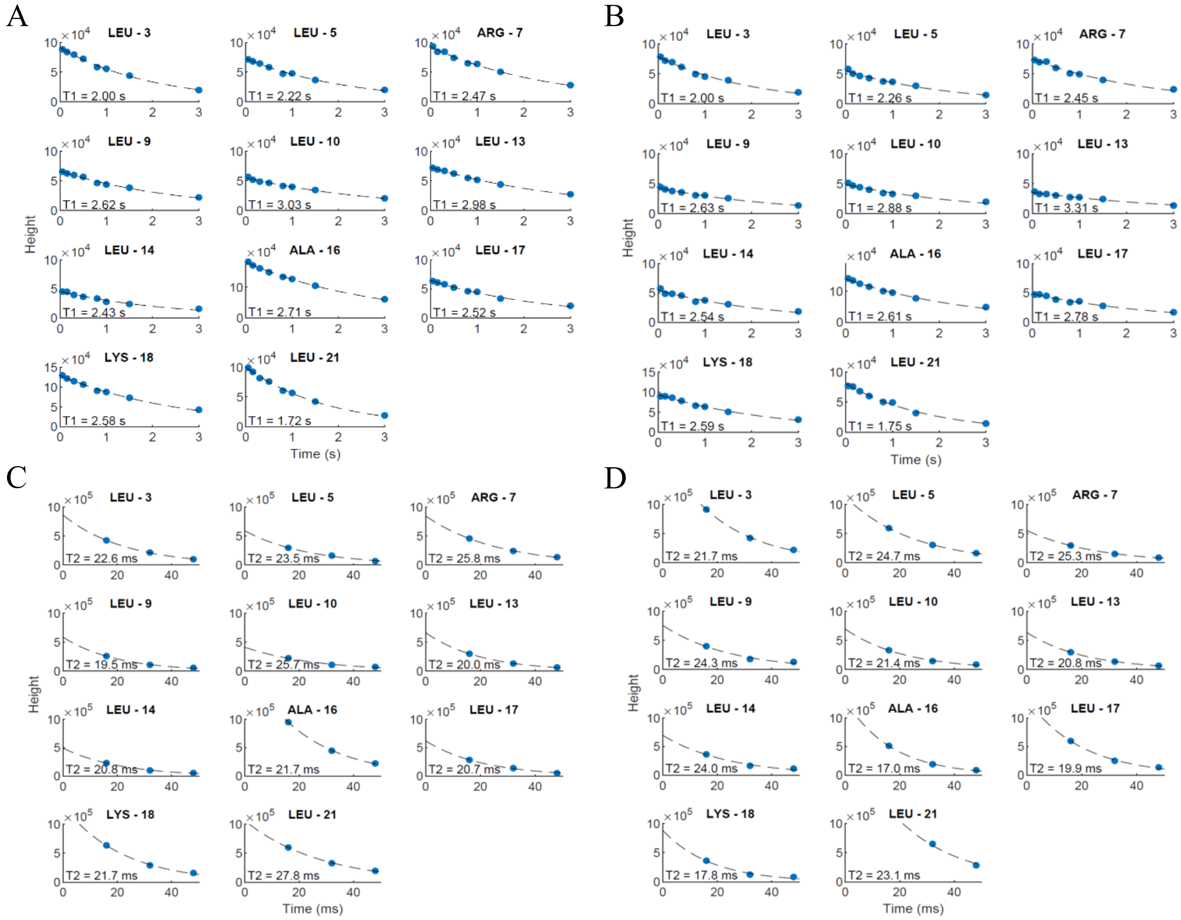

Figure S2. NMR relaxation parameter decays for the duplicate samples. (A) T_1 decays for sample 1. (B) T_1 decays for sample 2. (C) T_2 decays for sample 1. (D) T_2 decays for sample 2.

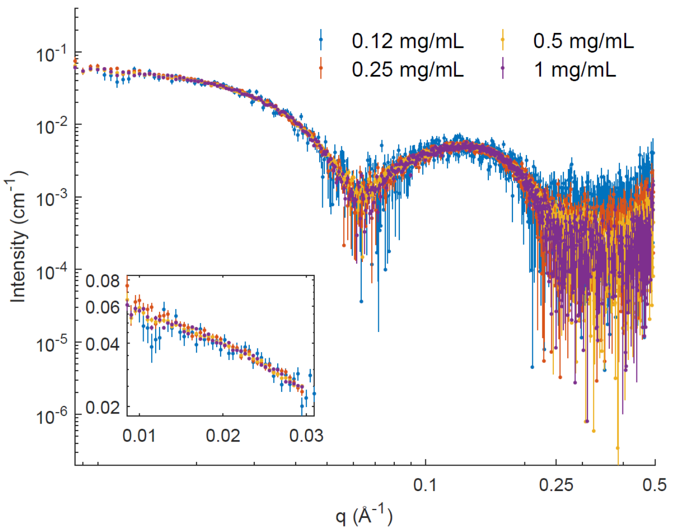

Figure S3. Influence of the peptide concentration in nanodiscs on the SAXS intensities. The insert is a zoom of the scattering intensities on the Guinier analysis region. No interparticle effect is observed.

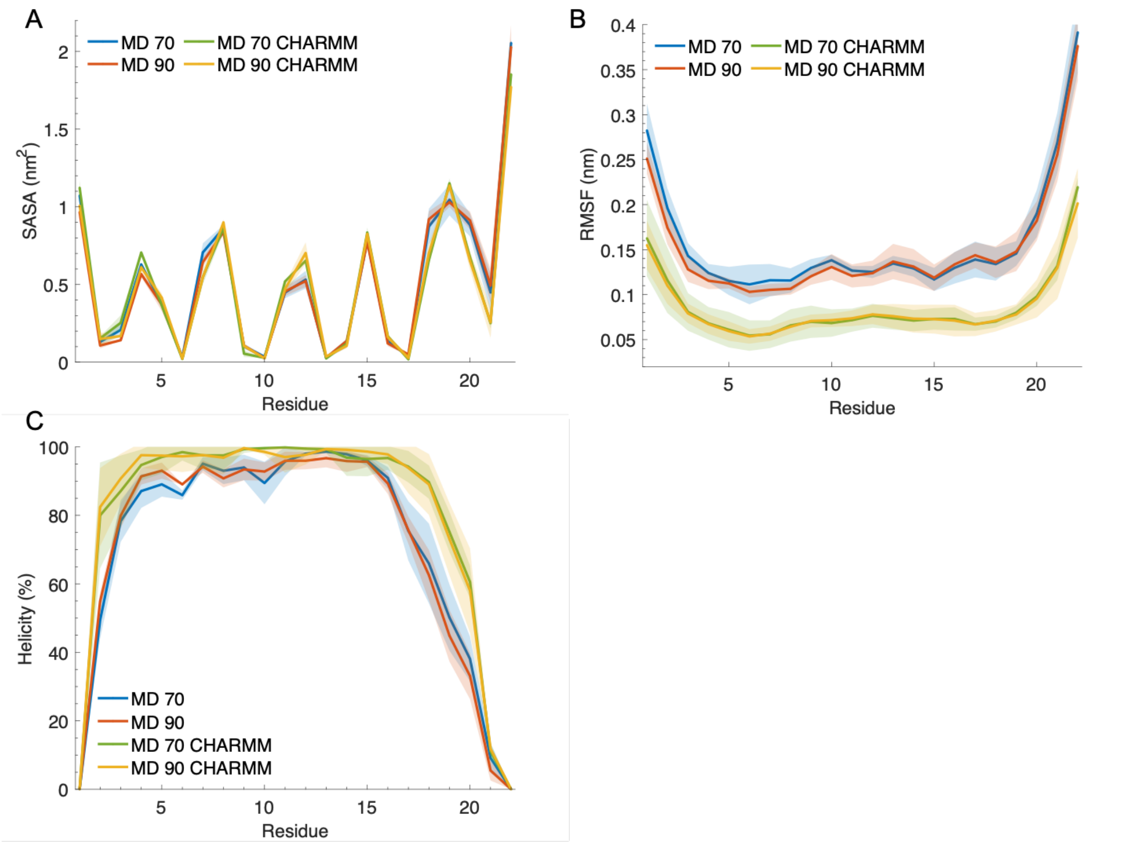

Figure S4. Peptide behavior under different force fields. (A) Solvent accessible surface area (SASA) per residue. (B) Root mean square fluctuation (RMSF) per residue. (C) Helicity per residue. MD data are represented as mean ± SD of triplicate simulations.

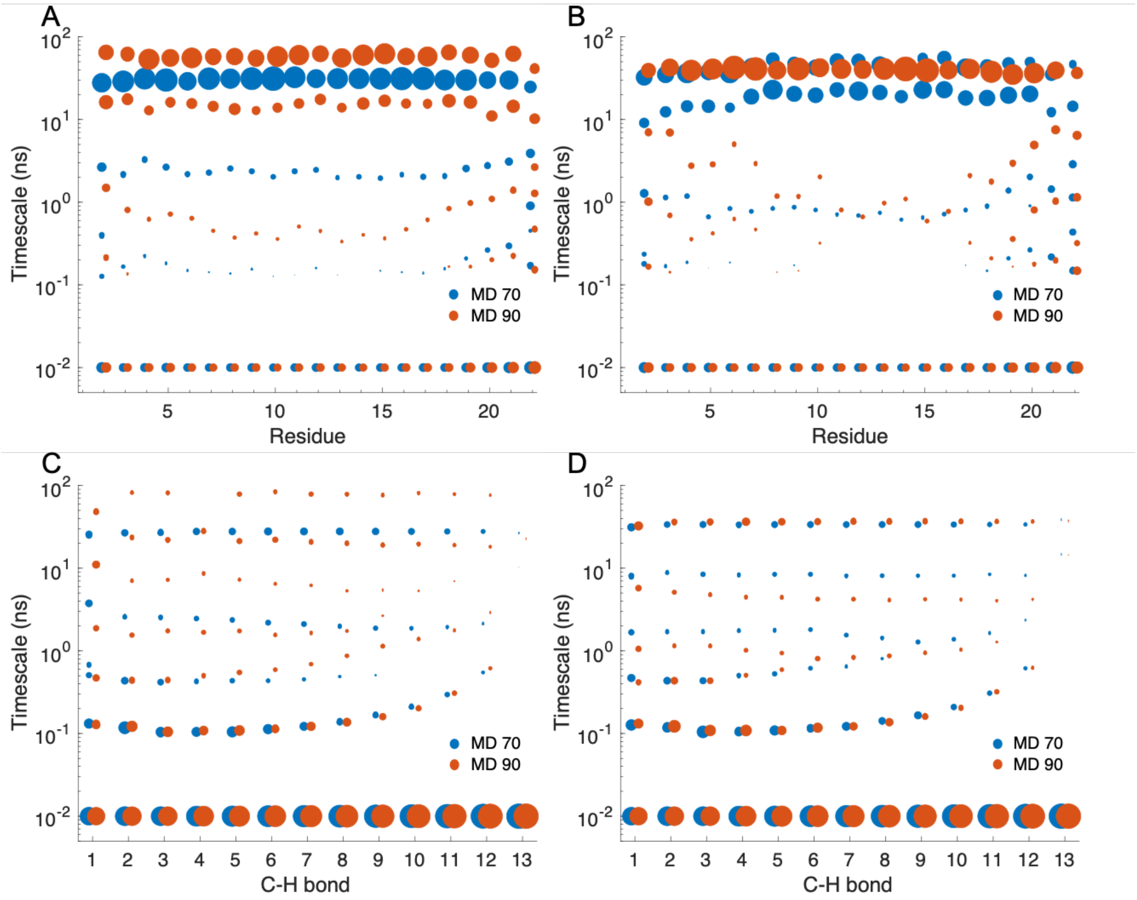

Figure S5. Dynamic landscape of 22A peptides and DMPC phospholipids. Dynamic landscapes of 22A peptides for the second replicate (A) and the third replicate (B). Dynamic landscapes of DMPC phospholipids for the second replicate (C) and the third replicate (D). C-H bond 13 refers to the terminal methyl group of DMPC phospholipids. The point sizes represent the weight of each timescale in the rotational relaxation process.

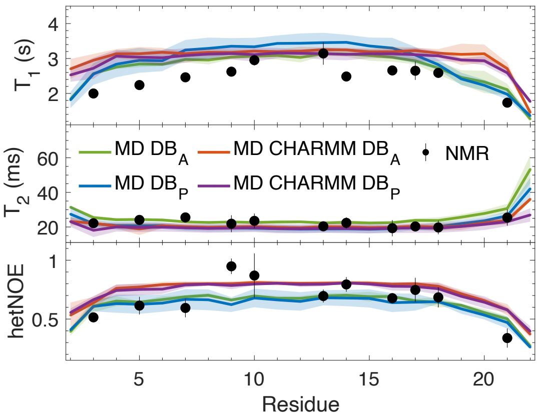

Figure S6. Spin relaxation times from different double belt (DB) arrangments and force fields. All MD simulations correspond to nanodiscs with 70 DMPC lipid molecules. The subscript A stands for a antiparallel configuration of peptides and P for a parallel configuration. MD data are represented as mean ± SD of triplicate simulations.

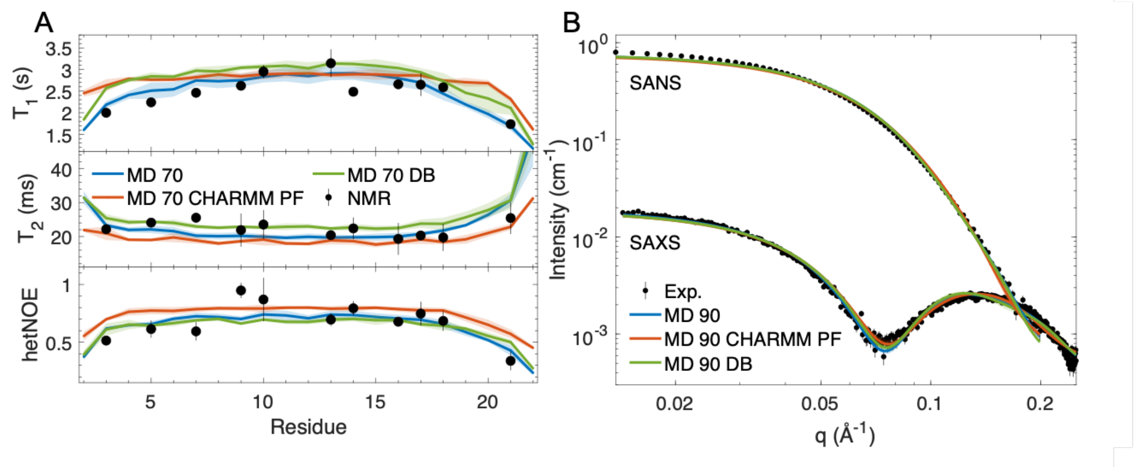

Figure S7. Effect of the orientation of peptides in the starting structure of nanodisc on the reproduction of experimental data from MD simulations. (A) NMR relaxation parameters. (B) SAXS and SANS curves. Peptides arrangements are in double belt (DB), picket fence (PF) or disordered. MD data are represented as mean ± SD of triplicate simulations.

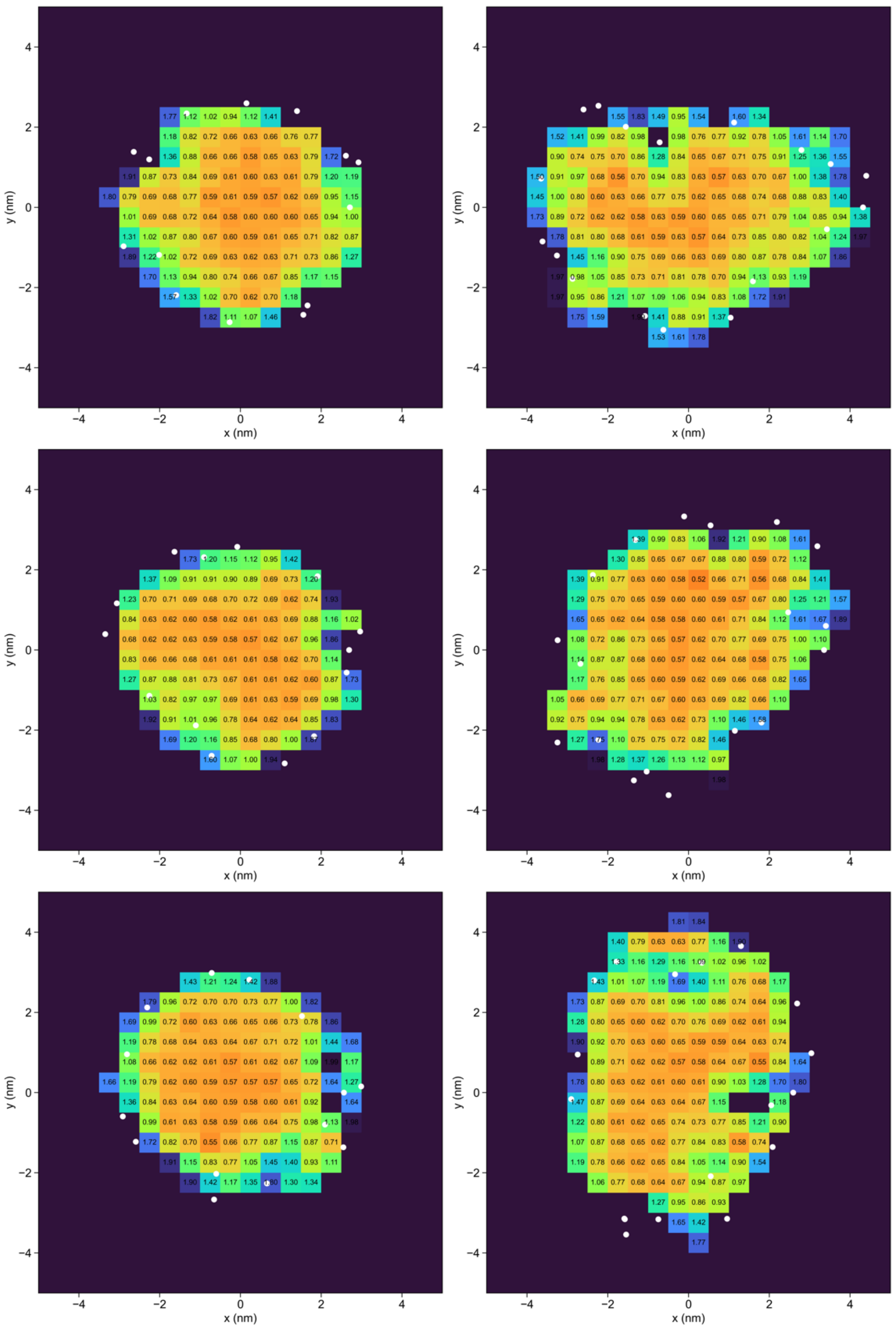

Figure S8. Area per lipid distributions. The plots for 70 (left) and 90 (right) AMBER system replicates are shown. Values are expressed as nm^2, and values above 2 nm^2 are not shown. The heatmap is colored from 0 to 2 nm^2. Average peptide centers of mass are shown as white points.

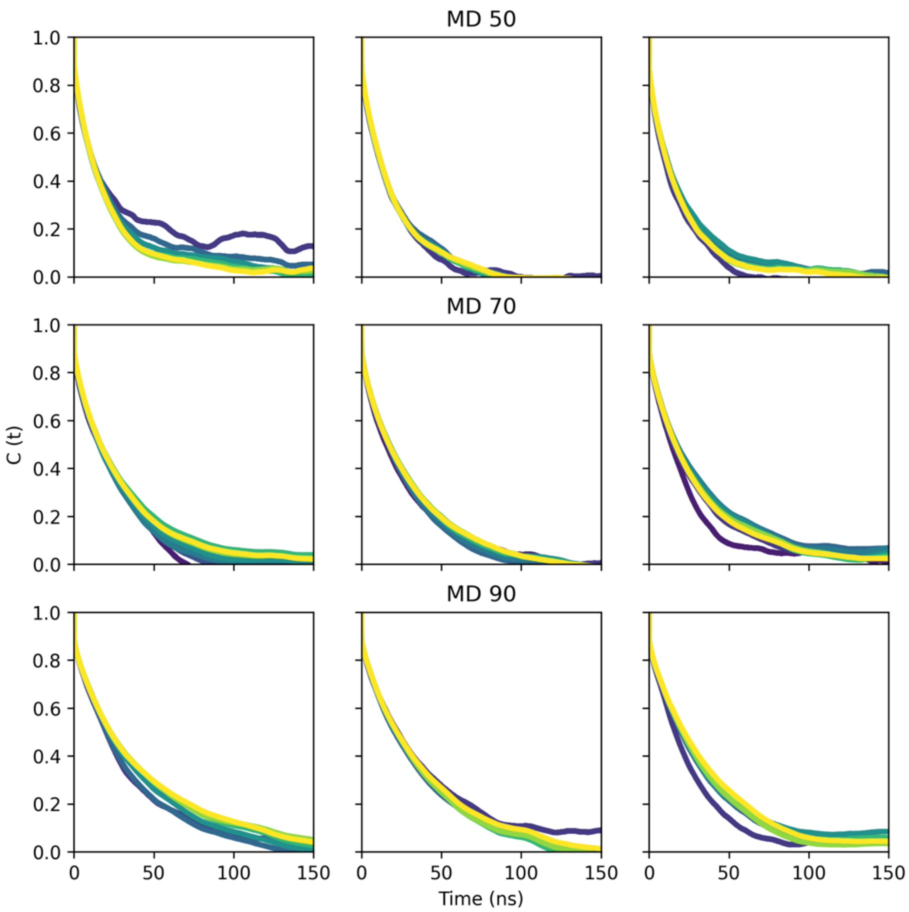

Figure S9. Rotational correlation function convergence of peptide residues 12 in AMBER systems started from MARTINI final snapshots. Line coloring represents the proportion of the trajectory used in calculating the correlation functions, starting from 0-0.5 μs (dark blue) and ending with 0-3 μs for MD 50 and 90, and 0-6 μs for MD 70 (light yellow).

Residue	δ_{1H} (ppm)	δ_{15N} (ppm)
LEU 3	8.94	121.17
LEU 5	7.76	121.10
ARG 7	8.14	118.90
LEU 9	8.46	119.06
LEU 10	8.44	121.58
LEU 13	8.29	118.87
LEU 14	8.33	120.77
ALA 16	7.98	121.98
LEU 17	8.13	118.20
LYS 18	8.38	119.31
LEU 21	7.70	119.65

Table S1. Assignment table of the labeled residues of 22A peptide in peptide nanodiscs. Chemical shifts of proton and nitrogen from the backbone amide groups of labeled residues are indicated in ppm.

Force field	Starting configuration	System size (number of lipids)	Box size in X, Y and Z dimensions	Length of rotacf analyzed	Simulated time and number of replicates
AMBER	MARTINI final snapshot	30	10 nm	50 ns	3 µs x 1
		50	11 nm	50 ns	3 µs x 3
		70	12 nm	100 ns	6 µs x 3
		90	14 nm	100 ns	3 µs x 3
		110	16 nm	100 ns*	3 µs + 1 µs x 2
	Parallel picket fence	70	12 nm	-	2 µs x 3
		90	14 nm	-	1 µs x 3
	Parallel double belt	70	12 nm	100 ns	3 µs x 3
	Antiparallel double belt	70	12 nm	100 ns	3 µs x 3
		90	14 nm	-	1 µs x 3
CHARMM	MARTINI final snapshot	70	12 nm	100 ns	3 µs x 3
		90	14 nm	-	1 µs x 3
	Parallel picket fence	70	12 nm	100 ns	3 µs x 3
		90	14 nm	-	1 µs x 3
	Parallel double belt	70	12 nm	100 ns	3 µs x 3
	Antiparallel double belt	70	12 nm	100 ns	3 µs x 3

*only the 3 µs trajectory was included in analysis

Table S2. Performed MD simulations. The number of lipids per system, the box size, the length of rotacf analyzed, the simulated time and number of replicates are indicated for the different simulations, depending on the force field used and the starting configuration.

	MD DB_A	MD DB_P	MD CHARMM DB_A	MD CHARMM DB_P
χ^2 - T1	0.61	0.81	1.03	0.88
χ^2 - T2	0.43	0.39	0.37	0.45
χ^2 - hetNOE	0.67	0.64	0.92	0.91

Table S3. Agreement between MD simulations and NMR experiments for nanodisc with peptide in double belt conformations in the starting structure for different force fields. The quality of the agreement between calculated and experimental data is reported as the χ^2 value.

	MD AMBER	MD CHARMM PF	MD DB_A
χ^2 - T1	0.29	0.59	0.61
χ^2 - T2	0.39	0.58	0.43
χ^2 - hetNOE	0.52	0.93	0.67
χ^2 - SAXS	3.54	8.46	6.20
χ^2 - SANS	4.80	8.35	5.79

Table S4. Agreement between MD simulations and experiments for different force fields and different orientations of peptides in the starting structure of nanodisc. The quality of the agreement between calculated and experimental data is reported as the χ^2 value.
