# Supplementary material for: Exploring the structure and dynamics of peptide nanodiscs through a synergistic approach with NMR spectroscopy, SAS and MD simulations
Source: Commun Chem. 2026 Apr 9;9:196. doi: 10.1038/s42004-026-02015-5 (PMC13247028; doi:10.1038/s42004-026-02015-5)
Supplement: Supplementary file 1 — Transparent Peer Review file [file 42004_2026_2015_MOESM1_ESM.pdf]

nature portfolio

Peer Review File

Exploring the structure and dynamics of peptide nanodiscs through a synergistic approach with NMR spectroscopy, SAS and MD simulations

Corresponding Author: Dr Sirine Nouri

This file contains all reviewer reports in order by version, followed by all author rebuttals in order by version.

Version 0:

Reviewer comments:

Reviewer #1

(Remarks to the Author)
COMMSCHEM-25-1215-T
In this manuscript, the authors investigate the structural and dynamical properties of 22A-nanodiscs using an integrated approach combining solution NMR spectroscopy, small-angle X-ray scattering (SAXS), small-angle neutron scattering (SANS), and molecular dynamics (MD) simulations. The major claim is that the central region of the 22A peptide forms a relatively rigid, ordered belt around the lipid bilayer, whereas the terminal regions, particularly the C-terminal K22 residue, remain conformationally disordered and solvent-exposed, potentially providing accessibility for interaction with LCAT and thereby offering a structural rationale for the functional competence of 22A nanodiscs.
Peptide-based nanodiscs are of broad and growing interest as alternatives to protein-based systems, and the multimodal methodology presented here will likely be valuable for studying other membrane mimetics. Overall, the conclusions are largely supported by the data, and the combined use of NMR, scattering, and simulations is a strength of the study, although a clearer discussion of NMR observables (e.g., peak intensities), inclusion of additional NMR data in the supplementary information, and comparison with previously characterized peptide nanodiscs would further strengthen the manuscript. With these refinements, the work has the potential to influence thinking in the field by improving understanding of how peptide sequence and terminal flexibility govern nanodisc structure and function, with implications for the rational design of peptide-based nanodiscs.

I have a few minor comments for the authors
1) The introduction and discussion would benefit from citing more recent literature on various peptide nanodiscs.

2) In the HSQC spectra, two distinct sets of cross-peaks are observed, differing substantially in intensity. Do these intensity variations reflect specific peptide-lipid interactions or heterogeneous dynamics within the nanodisc? Such intensity modulations have been reported for peptides interacting with binding partners (https://doi.org/10.1038/s42003-018-0063-1).

3) As the authors mentioned, "…room remains for improvement in experimental agreement…", the results are not very conclusive. Given that structures and properties of several peptide nanodiscs have been reported previously, the discussion should explicitly compare how peptide sequence variations influence structure, rigidity, and orientation relative to the plane of the lipid bilayer (https://doi.org/10.1074/jbc.M511475200).

4) The methods state that TOCSY and NOESY spectra were used for resonance assignment. These spectra, along with the complete chemical shift assignment table, should be included in the supplementary information. Additionally, have the authors compared NOESY spectra of the free peptide versus the nanodisc-bound peptide to probe peptide-lipid contacts and peptide orientation relative to the plane of the bilayer, as reported for other nanodisc-forming peptides (https://doi.org/10.1074/jbc.M511475200 & https://doi.org/10.1016/j.jcis.2023.09.162)?

5) In Figure 2A, the L21 (& L14) cross-peak appears overlapped with another signal. The presence of an additional peak is unexpected, as there is no metabolic scrambling for synthetically prepared peptides. Authors should comment on this. It would be helpful to include individual HSQC spectra of Leu-only-labelled and R7-A16-K18-labelled samples in the supplementary information, along with 1D projections of the relevant 2D cross-peaks.

6) Slightly increase the font size in all figures.

7) Use "…δ (ppm)" not "(ppm)" in NMR figure

Reviewer #2

(Remarks to the Author)
Manuscript by Nouri et al. reports the integrative structural study of peptide nanodiscs formed by 22A peptides. The authors suggest an interesting approach, in which several MD simulations are used to back-calculate the average parameters, such as NMR relaxation rates and scattering curves, and compare these parameters to the experimental values. Based on the good agreement of experimental and simulated data, the authors conclude on the composition and size of 22A nanodisc, and describe the parameters of 22A peptide packing in the rim of the particles.

Although the authors claim their motivation was to facilitate the design of novel therapeutics, I believe the proposed approach and the results are more interesting to structural biologists CryoEM microscopists, and NMR spectroscopists, especially those who may consider using peptide nanodiscs as a membrane mimetic for their proteins of interest. Altogether, the paper presents a novel approach and data that may provide a substantial improvement in the field of structural biology of membrane proteins. In my opinion, the paper should be published in Communications Chemistry after some corrections/additions.

Nevertheless, I have several comments:
1. I would suggest describing the whole concept of the approach in the first paragraph of the results question. Then it would be clear why the authors perform these particular experiments. As it is written now, the motivation for doing the experiments is sometimes unclear.
2. Line 105, SEC retention time of ~13 ml, what is the molecular weight (or hydrodynamic diameter) that this volume corresponds to?
3. On line 107 authors claim: "but we also observed heterogeneity". How was this heterogeneity observed? According to the authors, both DLS and SEC results correspond to the homogeneous solutions. This needs to be clarified.
4. line 116: Why were these particular three residues N/C labeled? This should be clarified in the text. It would also be helpful to have a sequence of the peptide in the results, at least in Figure 2.
5. line 121: How would the authors estimate the nanodisc size from T1/T2, using the model-free approach? Would the rotational correlation time from T1/T2 correspond to the rotational dynamics from the MD simulations and to the size distribution from DLS? Can one also derive a size distribution from the TEM images?
6. line397. What was the mixing time in the TOCSY experiment?
7. As one can see, the authors measured a 1H-NOESY experiment. In this regard, it would be interesting to look at the peptide-lipid contacts. Even if the lipids were deuterated, the headgroups are most likely still protonated and the profile of the peptide-lipid contacts could additionally support the model of the nanodisc, proposed by the authors.
8. line 353. The deuteration of DMPC (d54?) should be indicated in the materials and methods.
9. Based on the MD simulation, what could be said about the lipid packing in the nanodisc, compared to the bilayer? Can one observe a phase transition in 22A nanodisc? For example, by the 31P NMR, as described in the work (https://pubs.acs.org/doi/10.1021/acs.langmuir.7b03610).

Reviewer #3

(Remarks to the Author)
In the present paper the authors study the structure and dynamics of the apolipoprotein A-I mimetic peptide 22A nanodiscs by solution NMR, CD, TEM, DLS, SAXS, SANS, and MD-simulations approaches. At 1:5 peptide/lipid molar ratio nanodiscs are 8.4 nm size (DLS) and discoidal shape (TEM) with 22A peptide secondary structure being mostly helical (CD). I like the direct comparison of peptide nanodiscs with peptide-SDS micelles, where rigid nanodisc assembly is proven. It clearly demonstrated that in nanodiscs peptides and lipids rotate together as a rigid body. MD-simulation models with different number of lipid molecules per nanodisc were compared with SAXS, SANS and NMR experimental data. In the best MD model nanodiscs contain 90 lipids and 18 peptides on average.

However there are two sections which need improvement. At first, Introduction looks over-shortened to the point where it leads to a wrong impression for non-specialists. I address it in details in comment 1. The second section is 'Peptide orientation in nanodiscs from simulation-experiment integration' described in the comment 2. And the letter one will need additional MD-simulations to support the conclusion.

Overall the paper is well written and can be accepted for the publication after revision

1. Introduction. In the lines 73-74 you go with 'Results from solid-state NMR experiments of nanodisc forming peptides 14A and 18A on lipid bilayers suggested a double belt arrangement ...(15)' and this is over-shortened, taking into account that you compare it with ELK picket-fence MD-model with no clear experimental evidence (lines 75-76).
In the ref.15 14A and 18A peptide topology are directly observed from the oriented nanodiscs.
Right from the abstract of ref.15 "...Whereas at a peptide-to-lipid ratio of 2 mol% the peptides

align parallel to the bilayer surface, at 7.5 mol% disc-like structures are formed that spontaneously orient in the magnetic field of the NMR spectrometer. ... the detailed topology found in this work agrees with the peptides forming the rim of nanodiscs in a double belt arrangement." Nanodiscs are indeed kind of 'bilayers' but I would not call it like that in the present paper. Belt-like arrangement is therefore experimentally CONFIRMED for nanodiscs. However it is not clear if it's single belt, double belt, or even triple belt form. Therefore double belt arrangement is indeed SUGGESTED after double belt nanodiscs structure found in truncated apoA-I nanodiscs (ref.11) As a conclusion lines 73-74 are correct but misleading. Please reformulate it.
The same holds for lines 283-284.

2. 'Peptide orientation in nanodiscs from simulation-experiment integration' section.
22A peptide MD-simulation ends up with the model where 'substantial peptide ordering was not observed' (line 235). With a special efforts you managed to stabilize picket fence-like topology and compare with your experimental data (Figs S5, Table S2). However why haven't you even try to do the same for a belt-like peptide arrangement observed for 14A and 18A experimentally? This doesn't seem logic to me.
How I see it. Double-belt atomistic resolution structures of traditional nanodiscs – stabilized by membrane scaffold proteins (MSPs) was solved by NMR (ref.11). ApoA-I mimetic peptides 14A and 18A were shown to form belt arrangement by experimental NMR directly in the nanodiscs (ref.15). 'MD simulations suggested a picket fence like organization of ELK peptides at the peptide nanodisc rim (16, 17) but suitable data for experimental validation of such model is not available' (lines 75-76 in your manuscript). From this data set I would consider belt-like arrangement for 22A peptide nanodiscs.
Could you please complete the comparison with belt-like nanodiscs arrangements.
Recent paper on MD-simulations of 14A peptide nanodiscs where double-belt arrangement was observed with peptide dimer formation could be the one to compare with [BBA-Biomembranes 1868 (2026) 184495 doi.org/10.1016/j.bbamem.2025.184495]

3. Refs 18 and 28 are identical. Please correct

Version 1:

Reviewer comments:

Reviewer #1

(Remarks to the Author)
The authors have satisfactorily addressed all the comments and concerns raised during the review. I recommend acceptance of the manuscript for publication.

Reviewer #2

(Remarks to the Author)
I think the authors have adressed all of my concerns.

Reviewer #3

(Remarks to the Author)
The authors have significantly improved the paper and carefully addressed all the comments. I recommend to accept the paper as is.

Open Access This Peer Review File is licensed under a Creative Commons Attribution 4.0 International License, which permits use, sharing, adaptation, distribution and reproduction in any medium or format, as long as you give appropriate credit to the original author(s) and the source, provide a link to the Creative Commons license, and indicate if changes were made.
In cases where reviewers are anonymous, credit should be given to 'Anonymous Referee' and the source.
The images or other third party material in this Peer Review File are included in the article's Creative Commons license, unless indicated otherwise in a credit line to the material. If material is not included in the article's Creative Commons license and your intended use is not permitted by statutory regulation or exceeds the permitted use, you will need to obtain permission directly from the copyright holder.
To view a copy of this license, visit https://creativecommons.org/licenses/by/4.0/

Response to referees

Manuscript ID: COMMSCHEM-25-1215-T
Title: Exploring Peptide Nanodiscs Structure and Dynamics through Synergistic Approach of NMR spectroscopy, SAS and MD simulations.
Authors: Sirine Nouri, Akseli Niemelä, Ricky Nencini, Georgios Kolypetris, Tuomas Niemi-Aro, Salla I. Virtanen, O. H. Samuli Ollila, and Artturi Koivuniemi
Journal: Communications Chemistry

We thank the reviewers for their valuable comments and careful evaluation of our manuscript. We greatly appreciate their thorough assessment and constructive suggestions, which have helped us improve the quality of the work. The comments of the reviewers have been addressed in a point-by-point manner below and the changes made to the manuscript have been marked with red font. The comments of the reviewers are in black font and our answers are in blue font.

The main changes of the manuscript include:

- Clarification of the text and addition of new information and figures where requested
- New analysis of MD simulations with peptide oriented in a double belt configuration in the starting structure
- New analysis of the area per lipid distribution

We hope that, considering these substantial revisions and additional work, the manuscript is now suitable for publication in Communications Chemistry.

Reviewer 1

In this manuscript, the authors investigate the structural and dynamical properties of 22A-nanodiscs using an integrated approach combining solution NMR spectroscopy, small-angle X-ray scattering (SAXS), small-angle neutron scattering (SANS), and molecular dynamics (MD) simulations. The major claim is that the central region of the 22A peptide forms a relatively rigid, ordered belt around the lipid bilayer, whereas the terminal regions, particularly the C-terminal K22 residue, remain conformationally disordered and solvent-exposed, potentially providing accessibility for interaction with LCAT and thereby offering a structural rationale for the functional competence of 22A nanodiscs.

Peptide-based nanodiscs are of broad and growing interest as alternatives to protein-based systems, and the multimodal methodology presented here will likely be valuable for studying other membrane mimetics. Overall, the conclusions are largely supported by the data, and the combined use of NMR, scattering, and simulations is a strength of the study, although a clearer discussion of NMR observables (e.g., peak intensities), inclusion of additional NMR data in the supplementary information, and comparison with previously characterized peptide nanodiscs would further strengthen the manuscript. With these refinements, the work has the potential to influence thinking in the field by improving understanding of how peptide sequence and terminal flexibility govern nanodisc structure and function, with implications for the rational design of peptide-based nanodiscs.

I have a few minor comments for the authors:

1) The introduction and discussion would benefit from citing more recent literature on various peptide nanodiscs.

   We have now added more recent literature to the introduction and discussion regarding peptide-lipid nanodisc formulations (lines 53, 55, 388-390).

2) In the HSQC spectra, two distinct sets of cross-peaks are observed, differing substantially in intensity. Do these intensity variations reflect specific peptide-lipid interactions or heterogeneous dynamics within the nanodisc? Such intensity modulations have been reported for peptides interacting with binding partners (https://doi.org/10.1038/s42003-018-0063-1).

   We thank the reviewer for this insightful comment. The observed intensity differences between the two sets of cross-peaks could indeed arise from multiple factors, including specific peptide–lipid interactions and/or heterogeneous dynamics within the nanodisc. However, because of the complexity and heterogeneity of the systems, unambiguous discrimination between these possibilities would require additional dedicated and rather time-consuming experiments potentially complemented by additional biophysical methods. Although this is an interesting and important question, we believe that these experiments fall now beyond the scope of the present study.

3) As the authors mentioned, "…room remains for improvement in experimental agreement…", the results are not very conclusive. Given that structures and properties of several peptide nanodiscs have been reported previously, the discussion should explicitly compare how peptide sequence variations influence structure, rigidity, and orientation relative to the plane of the lipid bilayer (https://doi.org/10.1074/jbc.M511475200).

   We thank the reviewer for this important comment. We agree that, given the remaining deviations between simulations and experimental data, a more explicit discussion of the factors that may influence peptide configuration at the nanodisc rim is warranted. We have therefore expanded the discussion section to include a dedicated paragraph addressing how peptide length, charge distribution, conformational flexibility, and hydrophobic moment can modulate peptide orientation, inter-peptide interactions, and overall nanodisc organization (lines 365-382). We note, however, that these considerations remain hypothetical, as no currently available experimental method allows direct determination of peptide configuration ensembles at the nanodisc rim with atomic-level precision, and interpretations necessarily rely on integrative modeling approaches that we carried in this study.

4) The methods state that TOCSY and NOESY spectra were used for resonance assignment. These spectra, along with the complete chemical shift assignment table, should be included in the supplementary information. Additionally, have the authors compared NOESY spectra of the free peptide versus the nanodisc-bound peptide to probe peptide-lipid contacts and peptide orientation relative to the plane of the bilayer, as reported for other nanodisc-forming peptides (https://doi.org/10.1074/jbc.M511475200 & https://doi.org/10.1016/j.jcis.2023.09.162)?

   Table S1 has been added indicating the chemical shift assignment table of the imino protons for residues that were labeled in this study. We have the raw data of the TOCSY and NOESY spectra but unfortunately, the peak lists generated to perform the assignment are no longer available. This is because the assignment was done inside the Finnish Biological NMR center that has been closed in 2025. Neither the spectrometer nor the computer used to perform the assignment are accessible.

   We agree with the reviewer that it would be very interesting to probe peptide-lipid contacts in the NOESY to gain information on the peptide orientation relative to the plane of the

bilayer. We did measure a ^{15}N HSQC spectrum of a free peptide sample and we observed that the resolution of the peaks was really low, suggesting that aggregation was occurring (see purple spectrum in the following figure, in comparison with the green and blue spectra that represent 22A peptides in nanodiscs). Therefore, we did not measure other NMR experiment on the free peptide sample. We do see peaks in the region where intermolecular peptide/lipid NOE would be observed but we cannot know for sure that these peaks do not correspond to intramolecular peptide NOE.

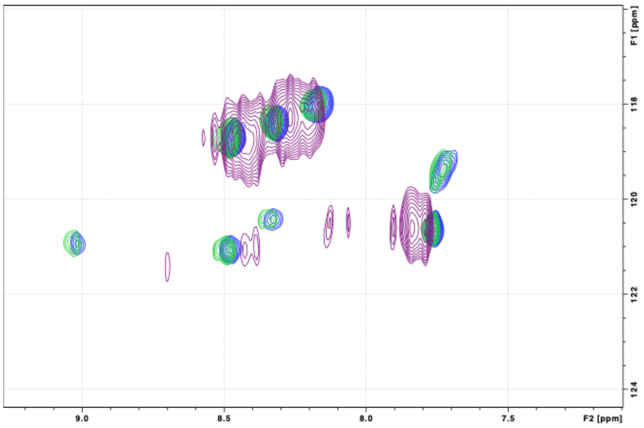

5) In Figure 2A, the L21 (& L14) cross-peak appears overlapped with another signal. The presence of an additional peak is unexpected, as there is no metabolic scrambling for synthetically prepared peptides. Authors should comment on this. It would be helpful to include individual HSQC spectra of Leu-only-labelled and R7-A16-K18-labelled samples in the supplementary information, along with 1D projections of the relevant 2D cross-peaks.

   We thank the reviewer for this comment as we do observe an overlap with another signal for L14 and L21 cross-peaks. Our hypothesis is that L14 and L21 might be in slightly different conformations in the multiple peptides in the rim of the nanodiscs, which would create a small shift in their chemical shifts and appear as overlapped signals.

   Figure S1 has been added to the supplementary information to show individual HSQC spectra and 1D projections.

6) Slightly increase the font size in all figures.

   The font size in all figures have been slightly increased.

7) Use "…δ (ppm)" not "(ppm)" in NMR figure.

   The labels in NMR figures have been changed according to this comment.

Reviewer 2

Manuscript by Nouri et al. reports the integrative structural study of peptide nanodiscs formed by 22A peptides. The authors suggest an interesting approach, in which several MD simulations are used to back-calculate the average parameters, such as NMR relaxation rates

and scattering curves, and compare these parameters to the experimental values. Based on the good agreement of experimental and simulated data, the authors conclude on the composition and size of 22A nanodisc, and describe the parameters of 22A peptide packing in the rim of the particles.

Although the authors claim their motivation was to facilitate the design of novel therapeutics, I believe the proposed approach and the results are more interesting to structural biologists CryoEM microscopists, and NMR spectroscopists, especially those who may consider using peptide nanodiscs as a membrane mimetic for their proteins of interest. Altogether, the paper presents a novel approach and data that may provide a substantial improvement in the field of structural biology of membrane proteins. In my opinion, the paper should be published in Communications Chemistry after some corrections/additions.

Nevertheless, I have several comments:

1) I would suggest describing the whole concept of the approach in the first paragraph of the results question. Then it would be clear why the authors perform these particular experiments. As it is written now, the motivation for doing the experiments is sometimes unclear.

   We thank the reviewer for this helpful suggestion. We agree that the conceptual framework of the study was not sufficiently introduced at the beginning of the results section. To improve clarity, we have now added a new introductory paragraph at the start of the results section that outlines the overall rationale, hypothesis, and research approach to resolve the structure of 22A peptide nanodiscs (lines 93-105).

2) Line 105, SEC retention time of ~13 ml, what is the molecular weight (or hydrodynamic diameter) that this volume corresponds to?

   To quantify the peak observed in SEC, we collected 10 fractions of 500 µL over the peak and we measured the hydrodynamic diameter of the fractions with DLS. Over the 10 fractions, we obtained a mean hydrodynamic diameter of 8.8 nm with a standard deviation of 1.2 nm, confirming that the observed peak in SEC corresponds to the nanodiscs.

3) On line 107 authors claim: "but we also observed heterogeneity". How was this heterogeneity observed? According to the authors, both DLS and SEC results correspond to the homogeneous solutions. This needs to be clarified.

   Our DLS and SEC results show that we have a homogeneous solution of nanodiscs as we do not observe free peptides or free lipids in solution. However, according to the relatively large width of the peaks in DLS and SEC, we think that there is polydispersity in the size of the nanodiscs in the sample. To clarify this point, the word "heterogeneity" as being replaced with the word "polydispersity" in the text (line 118).

4) Line 116: Why were these particular three residues N/C labeled? This should be clarified in the text. It would also be helpful to have a sequence of the peptide in the results, at least in Figure 2.

   We chose to labeled three extra residues to have more experimental NMR data throughout the sequence. Those three residues were chosen for economic reasons as they were the less expensive and because they had distinct ^{15}N chemical shift ranges from each other to facilitate the assignment. Also, one of our motivations was to measure paramagnetic

relaxation enhancement (PRE) to probe the orientation of the peptide as done in this paper (https://pubs.acs.org/doi/10.1021/ja069004f). We chose to labeled ARG 7 and LYS 18 because they were predicted not to face the lipid bilayer. The measured PRE were too small to obtain reliable results for this paper. A sentence has been added in the Materials and Methods section to clarify this point (lines 398-399).

The sequence of the peptide has been added to Figure 2.

5) Line 121: How would the authors estimate the nanodisc size from T1/T2, using the model-free approach? Would the rotational correlation time from T1/T2 correspond to the rotational dynamics from the MD simulations and to the size distribution from DLS? Can one also derive a size distribution from the TEM images?

   We thank the reviewer for the suggestion to employ the model-free approach to estimate nanodisc size. The analysis has been included in the main text (lines 285-290). Briefly, our simulations indicate that the dominant rotational timescales for peptides are at ~30 ns, while the model-free approach gives a value of ~26 ns. A likely reason for the difference is the non-spherical shape of the nanodiscs. Using the value of ~30 ns from the 70 lipid MD systems, the Stokes-Einstein-Debye relation gives a diameter of 7.1 nm. This is slightly smaller than the diameter calculated with Hydropro10 software, and the approximate mean from DLS. Again, the value is likely affected by the assumption of sphericity.

   We used the software ImageJ to calculate the size distribution from the TEM images but unfortunately the contrast between the nanodiscs and the background was too low to successfully perform this analysis.

6) Line 397: What was the mixing time in the TOCSY experiment?

   The mixing time in the TOCSY experiment was 60 ms. This information has been added to the text (line 445).

7) As one can see, the authors measured a 1H-NOESY experiment. In this regard, it would be interesting to look at the peptide-lipid contacts. Even if the lipids were deuterated, the headgroups are most likely still protonated and the profile of the peptide-lipid contacts could additionally support the model of the nanodisc, proposed by the authors.

   We agree with the reviewer that investigating the profile of peptide-lipids contacts should give information on the peptide orientation relative to the plane of the bilayer. As we used deuterated DMPC, only the headgroups are protonated. If the peptides were arranged in a picket fence configuration, we should observe peptide/lipid NOE cross-peaks between the lipid headgroups and the imino protons of residues at the beginning or at the end of the peptide sequence. The resonances of lipid headgroup protons have chemical shifts between 3.8 and 4.5 ppm (https://doi.org/10.1074/jbc.M511475200). They overlap with the resonances of side chain protons of the peptides. Thus, it is really difficult with only one ^{1}H-NOESY experiment to distinguish intramolecular peptide NOE and peptide-lipid NOE cross-peaks. To solve this problem, we would need to prepare a sample with fully deuterated DMPC lipids, measure a ^{1}H-NOESY experiment and identify which peaks are missing from the ^{1}H-NOESY experiment measured with d54 DMPC lipids.

8) Line 353: The deuteration of DMPC (d54?) should be indicated in the materials and methods.

The deuteration of DMPC has been added to the materials and methods (line 399).

9) Based on the MD simulation, what could be said about the lipid packing in the nanodisc, compared to the bilayer? Can one observe a phase transition in 22A nanodisc? For example, by the 31P NMR, as described in the work (https://pubs.acs.org/doi/10.1021/acs.langmuir.7b03610).

We have now performed area per lipid distribution analysis for the systems (Figure S8, lines 296-298). In general, the nanodisc center is more packed than the perimeter with an area per lipid of ~0.60 nm^2. However, the perimeter distribution is affected by the dynamic morphology of the nanodiscs, peptide occlusion and the plane fitting algorithm so accurate assessment is difficult.

We performed ^{31}P NMR experiments to monitor phase transition on nanodisc samples at different temperatures and with different lipid compositions. In our NMR facility, the 850 MHz spectrometer that we used to perform the NMR experiments in this study was equipped with a TCI CryoProbe that did not allow us to perform ^{31}P NMR experiment. We had to use a SmartProbe BBFO on a 600 MHz spectrometer. However, at this magnetic field and without a cryoprobe, the resolution of the ^{31}P NMR peaks was not good enough to characterize phase transition in our samples.

Reviewer 3

In the present paper the authors study the structure and dynamics of the apolipoprotein A-I mimetic peptide 22A nanodiscs by solution NMR, CD, TEM, DLS, SAXS, SANS, and MD-simulations approaches. At 1:5 peptide/lipid molar ratio nanodiscs are 8.4 nm size (DLS) and discoidal shape (TEM) with 22A peptide secondary structure being mostly helical (CD). I like the direct comparison of peptide nanodiscs with peptide-SDS micelles, where rigid nanodisc assembly is proven. It clearly demonstrated that in nanodiscs peptides and lipids rotate together as a rigid body. MD-simulation models with different number of lipid molecules per nanodisc were compared with SAXS, SANS and NMR experimental data. In the best MD model nanodiscs contain 90 lipids and 18 peptides on average.

However there are two sections which need improvement. At first, Introduction looks over-shortened to the point where it leads to a wrong impression for non-specialists. I address it in details in comment 1. The second section is 'Peptide orientation in nanodiscs from simulation-experiment integration' described in the comment 2. And the letter one will need additional MD-simulations to support the conclusion. Overall the paper is well written and can be accepted for the publication after revision.

1) Introduction. In the lines 73-74 you go with 'Results from solid-state NMR experiments of nanodisc forming peptides 14A and 18A on lipid bilayers suggested a double belt arrangement ...(15)' and this is over-shortened, taking into account that you compare it with ELK picket-fence MD-model with no clear experimental evidence (lines 75-76). In the ref.15 14A and 18A peptide topology are directly observed from the oriented nanodiscs. Right from the abstract of ref.15 "...Whereas at a peptide-to-lipid ratio of 2 mol% the peptides align parallel to the bilayer surface, at 7.5 mol% disc-like structures are formed that spontaneously orient in the magnetic field of the NMR spectrometer. ... the detailed topology found in this work agrees with the peptides forming the rim of nanodiscs in a double belt arrangement." Nanodiscs are indeed kind of 'bilayers' but I would not call it like that in the present paper. Belt-like arrangement is therefore experimentally CONFIRMED for nanodiscs. However it is not clear if it's single belt, double belt, or even triple belt form. Therefore double belt arrangement is indeed SUGGESTED after double belt nanodiscs

structure found in truncated apoA-I nanodiscs (ref.11) As a conclusion lines 73-74 are correct but misleading. Please reformulate it. The same holds for lines 283-284.

We thank the reviewer for pointing this out. Indeed, our use of the term “bilayer” was misleading and we have now corrected it (lines 71 and 310).

2) 'Peptide orientation in nanodiscs from simulation-experiment integration' section. 22A peptide MD-simulation ends up with the model where 'substantial peptide ordering was not observed' (line 235). With a special efforts you managed to stabilize picket fence-like topology and compare with your experimental data (Figs S5, Table S2). However why haven't you even try to do the same for a belt-like peptide arrangement observed for 14A and 18A experimentally? This doesn't seem logic to me. How I see it. Double-belt atomistic resolution structures of traditional nanodiscs – stabilized by membrane scaffold proteins (MSPs) was solved by NMR (ref.11). ApoA-I mimetic peptides 14A and 18A were shown to form belt arrangement by experimental NMR directly in the nanodiscs (ref.15). 'MD simulations suggested a picket fence like organization of ELK peptides at the peptide nanodisc rim (16, 17) but suitable data for experimental validation of such model is not available' (lines 75-76 in your manuscript). From this data set I would consider belt-like arrangement for 22A peptide nanodiscs. Could you please complete the comparison with belt-like nanodiscs arrangements. Recent paper on MD-simulations of 14A peptide nanodiscs where double-belt arrangement was observed with peptide dimer formation could be the one to compare with [BBA-Biomembranes 1868 (2026) 184495 doi.org/10.1016/j.bbamem.2025.184495]

We thank the reviewer for noticing this discrepancy. Originally, we had attempted to start simulations from an ideal double belt configuration. However, with the determined peptide:lipid ratio of 1:5 the peptide pairs formed too large a circle for the lipids to fill properly. We now opted to tighten the circle by relocating peptides as needed to partial triple belt configurations. We ran systems with AMBER and CHARMM force fields in both parallel and antiparallel double belt configurations for 3 µs. As the AMBER antiparallel attained best fitting values to experiments we simulated its 90 system as well to get SAXS and SANS values. Overall, the fitting was worse than a system started with a random configuration (Figure S7, Table S4), but it was a valuable addition to the manuscript. Like with the picket fence systems these simulations seem to suggest that a disordered peptide configuration fits with experimental data better, but we cannot conclusively rule out the more ordered structures either. This analysis has been included in the main text (lines 262-268).

3) Refs 18 and 28 are identical. Please correct.

We thank the reviewer for noticing this mistake. It has now been corrected.
